# Complete mitochondrial genome sequencing of *Oxycarenus laetus* (Hemiptera: Lygaeidae) from two geographically distinct regions of India

**DOI:** 10.1038/s41598-021-02881-0

**Published:** 2021-12-09

**Authors:** Shruthi Chalil Sureshan, Ruchi Vivekanand Tanavade, Sewali Ghosh, Saswati Ghosh, Raja Natesan Sella, Habeeb Shaik Mohideen

**Affiliations:** 1 Bioinformatics and Entomoinformatics Lab, Department of Genetic Engineering, School of Bioengineering, SRM Institute of Science and Technology, Kattankulathur, 603203 Tamil Nadu India; 2 Department of Advanced Zoology and Biotechnology, Guru Nanak College, Chennai, 600042 Tamil Nadu India; 3 Department of Virology, King Institute of Preventive Medicine and Research, Chennai, 600032 Tamil Nadu India; 4 Membrane Protein Lab, Department of Genetic Engineering, School of Bioengineering, SRM Institute of Science and Technology, Kattankulathur, 603203 Tamil Nadu India

**Keywords:** Computational biology and bioinformatics, Evolution

## Abstract

*Oxycarenus laetus* is a seed-sap sucking pest affecting a variety of crops, including cotton plants. Rising incidence and pesticide resistance by *O. laetus* have been reported from India and neighbouring countries. In this study, *O. laetus* samples were collected from Bhatinda and Coimbatore (India). Pure mtDNA was isolated and sequenced using Illumina MiSeq. Both the samples were found to be identical species (99.9%), and the complete genome was circular (15,672 bp), consisting of 13 PCGs, 2 rRNA, 23 tRNA genes, and a 962 bp control region. The mitogenome is 74.1% AT-rich, 0.11 AT, and − 0.19 GC skewed. All the genes had ATN as the start codon except cox1 (TTG), and an additional *trnT* was predicted. Nearly all tRNAs folded into the clover-leaf structure, except *trnS1* and *trnV*. The intergenic space between *trnH* and *nad4*, considered as a synapomorphy of Lygaeoidea, was displaced. Two 5 bp motifs AATGA and ACCTA, two tandem repeats, and a few microsatellite sequences, were also found. The phylogenetic tree was constructed using 36 mitogenomes from 7 super-families of Hemiptera by employing rigorous bootstrapping and ML. Ours is the first study to sequence the complete mitogenome of *O. laetus* or any *Oxycarenus* species. The findings from this study would further help in the evolutionary studies of Lygaeidae.

## Introduction

Polyphagous seed feeding dusky cotton bug *Oxycarenus laetus* infects various crops, especially cotton plants and the Malvaceae family^1^. Its incidence is on the rise, and it is reported from 100 + countries^2,3^. The life duration of this insect lasts from 35 to 45 days through five nymphal stages to develop into a fully grown adult^4^. Reports suggest the incidence of secondary pests is steeply increasing on cotton, especially the *Oxycarenus spp*.^5^, including on the Bt varieties. The trend is similar in nearby China and Pakistan^6^; it has become resistant to several pesticides, and synergistic actions were required to control it^7^.

Mitochondrial DNA has been a very significant marker in population and evolutionary studies. Uniparental transmission to offspring, high copy number per cell, and susceptibility to rapid evolution render mtDNA highly variable^8^. Though resistance to arthropods due to mtDNA is yet to be ascertained, several studies have reported cryptic species as one of the reasons; arising due to mutations either in the mtDNA or nuclear DNA. Uniparental maternal inheritance involving mtDNA has been reported as one of the reasons for the difference between susceptible and resistant species^9^. Recently, Ding et al.^10^, by employing mitochondrial genome and transcriptome sequencing, identified the role of mitochondrial genes *atp8* and *nad5* and the associated amino acid mutations to be rendering resistance to pyrethroid by *A. sinensis* population.

Due to its conserved nature and critical genetic markers, mtDNA has become the primary source for phylogenomics^11^. Identification of insects is crucial to managing endangered, protected, and invasive species in the ecosystem^12^. Insect mitogenome size ranges from 14,503 to 19,517 bp^13,14^. Most insects code for typical 37 genes, including 13 protein coding genes (PCGs), 22 tRNA genes and 2 rRNA genes, and a control region^15^. The most prominent non-coding region is the control region, also called the A + T rich region, responsible for transcription and translation. It is assumed to be analogous to the *oriC*^16^. Despite the technological advancements in biotechnology, there are only 9046 insect mitogenomes, of which 1016 are from the order Hemiptera and only four from its Lygaeidae family (as of 26/06/2020) which provides much scope in insect mitogenomic studies.

It has been found that the North Indian fields had a higher incidence and resistance to pesticides by this bug in comparison to the South Indian cotton fields^7,17,18^. It could be due to the existence of different/cryptic species altogether or due to mutations in the detoxification system. In this study, the mitogenome of *O. laetus* (Bhatinda, N. India (BTI) and Coimbatore, S. India (CBE)) was wholly sequenced and annotated, making it the first such study in the genus *Oxycarenus*. Samples are collected from two locations that differ drastically in climate, soil conditions, and geography. This study was done to understand if two different species inhabit these regions. Studying the mitogenome of this insect could open up new avenues in Hemiptera research and its phylogenomics and better pest control.

## Materials and methods

### Sample collection

Samples of *O. laetus* were collected from the post harvested cotton fields located in Punjab Agriculture University, BTI (Punjab), and Tamilnadu Agriculture University, CBE (Tamilnadu) in India, with the help of scientists (species confirmation) and staff. Cotton bolls carrying *O. laetus* were plucked and transferred to transparent plastic boxes with their mouths covered with muslin clothe and transported to the laboratory. They were reared until an ample population was raised to extract mtDNA. Insects were reared on cotton bolls and maintained at 16:8 h of light:dark photoperiod, 27 °C, and 55% humidity. All the experiments were performed on the F1 generation population. All the chemicals and reagents used in this study were of finite grade.

### Extraction and validation of mitochondrial DNA

A mixed population of adult *O. laetus* weighing 200 mg from both locations was macerated in 10 ml of homogenization buffer (0.4 M sucrose, 50 mM Tris HCl pH 7.5, 50 mM EDTA, 1% BSA, 10 mM β-mercaptoethanol)^19^. The mixture was centrifuged at 3500 rpm for 5 min at 4 °C. The supernatant was collected and centrifuged twice to remove cell debris and nuclear DNA. The supernatant was then centrifuged at 14,000 rpm for 15 min to pellet the mitochondria and resuspended in DNase-RNase free water. Traces of nuclear DNA were removed by incubating the mixture in 2.5 U of DNase I and DNase buffer (Qiagen) for 30 min. The action of DNase was terminated by adding 30 μl of 0.5 M EDTA. The mixture was again centrifuged at 12,000 rpm for 5 min, and the pellet was resuspended in 500 µl lysis buffer (100 mM Tris pH 8, 10 mM EDTA, 2% SDS, 15 μl of 20 mg/ml proteinase K), and the mixture was incubated at 55 °C for 1 h.

Then, 140 μl of 5 M NaCl and 250 μl of 2% CTAB were added to the mixture and incubated at 62 °C for 25 min. RNA contamination was removed by adding 10 μl of 20 mg/mL RNase A and incubation at 37 °C for one hour. An equal volume of chloroform:isoamyl alcohol (24:1) was added to the mixture and centrifuged at 14,000 rpm for 15 min. The top aqueous phase was collected, and the step was repeated. The final aqueous phase was collected, and two volumes of isopropanol was added and incubated at 20 °C for one hour. The mtDNA was precipitated by centrifugation at 14,000 rpm for 15 min and washed with 70% ethanol. The air-dried pellet was then dissolved in 20–30 μl of TE buffer and quantitated using NanoDrop.

The purity of the isolated mitochondrial DNA was assessed by amplifying COI (mitochondrial genome-specific) and Histone 4 gene (nuclear genome-specific). Table 1 shows the primers used in this study. The following conditions were used to amplify the COI gene: initial denaturation at 94 °C for 5 min; 30 cycles of final denaturation at 94 °C for 1 min, annealing at 52 °C for 1 min, elongation at 72 °C for 2 min 30 s; and final elongation at 72 °C for 5 min. Conditions used for amplifying Histone 4 gene are as follows: initial denaturation at 94 °C for 3 min; followed by 30 cycles of final denaturation at 94 °C for 1 min, annealing at 52 °C for 1 min, elongation at 72 °C for 1 min; and final elongation for 5 min. PCR products were visualized on 1% agarose gel.

**Table 1:** Primers used in the study.

Name	Sequence
COI-forward	GGTCAACAAATCATAAAGATATTGG
COI-reverse	TAAACTTCAGGGTGACCAAAAAATCA
Histone4-forward	ATTTCCACTCTGGTGGATAAGC
Histone4-reverse	ACACTTGGGCCTTTTAACTTTG
tRNH-NADH5-gap-forward	TTTCCCAATCAACAAAATAAAAA
tRNH-NADH5-gap-reverse	TTGGGTCATTCTTTTTCAGG

### Mitogenome sequencing and assembly

Nextera XT DNA Library preparation protocol was followed for library preparation using 1 ng of fragmented and tagged quantified DNA. The DNA was tagged using Amplicon Tagment Mix present in the Nextera XT kit. The DNA was then subjected to 12 cycles of indexing-PCR, and the product was purified by high-prep magnetic beads and quantified. The DNA samples were sequenced De novo using MiSeq Illumina sequencer to generate paired-end reads of length 75 bp. Raw data generated in this study were submitted to NCBI’s SRA Archives (PRJNA520830). The raw reads were proofread using FastQC^20^, and the low-quality reads and adapter sequences were filtered using Trimmomatic (0.38 v)^21^ and Cutadapt (2.8v)^22^. The high-quality reads were assembled using NOVOPlasty^23^.

### Sequence analysis, annotation, and visualization

MITOS-1 and MITOS-2^24^ were used to annotate the assembled mitogenome. The PCGs were confirmed by using ORF Finder, and BlastN confirmed rRNA and tRNAs. The control region was validated by aligning it with the transcriptome of data generated in our previous study (SRX5329347) using standalone BLAST. MEGA 7.0^25^ was used to determine nucleotide composition, codon usage, and relative synonymous codon usage (RSCU). Skewness in the mitogenome was calculated using the formula: AT-skew = (A − T)/(A + T); GC-skew = (G − C)/(G + C)^26^. Tandem repeats in the mitogenome were identified using Tandem Repeats Finder^27^. Secondary structures of tRNA were predicted using Mfold^28^. The CGView Server^29^ was used to generate the circular map of the mitogenome.

### Cross verification of the rearrangement of intergenic region

The rearrangement of the conserved intergenic region of Lygaeidae was verified by designing primers to target and amplify the specific spacer. The amplified product was sequenced using the Sanger method and aligned with the assembled mitogenome. Table 1 lists the primer pair used for verification. PCR conditions used were as follows: initial denaturation at 94 °C for 3 min; 30 cycles of denaturation at 94 °C for 1 min, annealing at 57 °C for 1 min, elongation at 72 °C for 1 min; and final elongation at 72 °C for 5 min (Fig. S1).

### Phylogenetic analysis

The phylogenetic study was done using 35 other species from hemipteran super-families. These included Aphidoidea, Cimicoidea, Reduvioidea, Pentatomoidea, Coreoidea, Pyrrhocoroidea, and Lygaeoidea (Table 4). Similar methodology as was followed by^30–32^ was employed with few changes as was needed for this study. The significant difference here was that we used whole mitogenomes instead of PCGs, intending to study the relationship among the selected data as a function of complete mitogenome. Both PhyML online server^33^ and raxMLGUI^34^ were used to construct the phylogenetic tree. The mitogenomes were aligned using MUSCLE, conserved regions were identified, and the unreliable regions in the data were removed using the Gblocks program. In the raxmlGUI, ML + bootstrapping analysis was done involving 1000 replications invoking the GTRGAMMAI substitution model. The resulting tree was visualized using TreeDyn. We also used the Bayesian Inference method to generate a phylogenetic tree in MrBayes v3.2.7 in order to validate the tree^35^. Markov chain Monte Carlo (MCMC) simulations were run involving 1,000,000 generations with sampling every 1000 generations and the process was continued until the average standard deviation of split frequencies < 0.01. The first 25% sample trees were discarded in the burn-in process and the remaining trees were used to form the consensus tree and posterior probability (PP) values.

## Results

### Genome organization and structure of *O. laetus*

The MiSeq data of BTI and CBE samples of *O. laetus* was submitted to the NCBI SRA database under the accession numbers SRR11024516 and SRR11024517, respectively. The study was done to understand whether there exists a different species of the genus *Oxycarenus* similar to *O. laetus*, which might be the reason for higher incidence and resistance. The study results are such that the mitogenomes of both the samples were highly identical (99.9%), confirming both to be identical species. Therefore, to maintain clarity of reading, findings of only one sample will be discussed hereafter, unless mentioned elsewhere in the manuscript.

**Figure 1:**
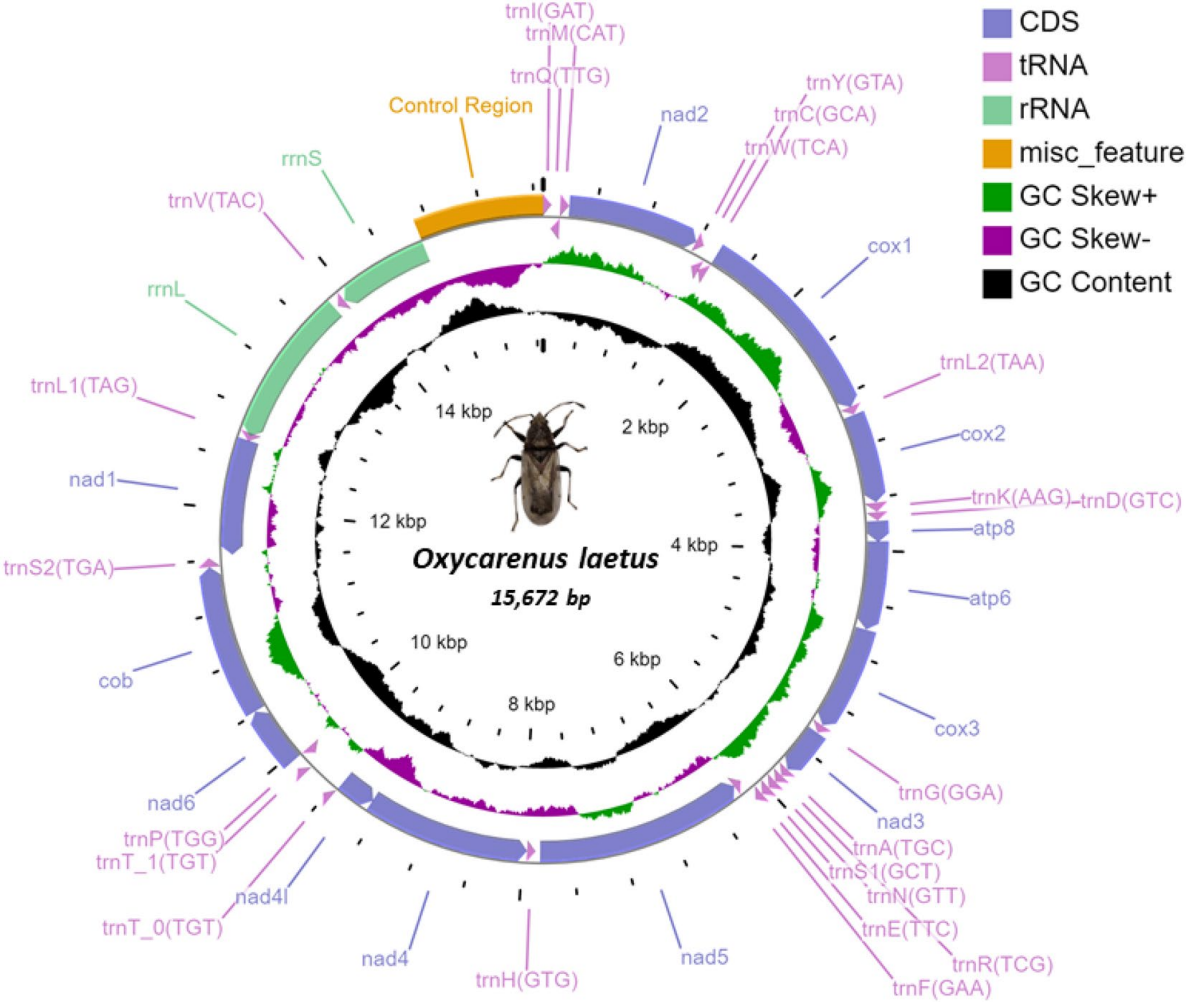
Circular map of the mitogenome of *O. laetus*. The above figure tells us the location of PCGs, tRNAs, rRNAs, and control region. The inner green, purple and black circles show the -ve GC skew, + ve GC skew, and GC content.

The mitogenome of *O. laetus* had all the general characteristics of other similar insects. The 15,672 bp long mitogenome is double-stranded and circular, consisting of typical 13 PCGs, 2 rRNA, and 23 tRNA genes. A unique feature witnessed was 38 genes instead of the typical 37 genes and a control region spanning 962 bp. The majority J-strand had 24, and the minority N-strand harbours 14 of these 38 genes. Gene overlaps were observed at 16 locations, and the longest overlap involved 25 bp between *trnL1* and *rRNAL*^36^. Two overlaps found between *atp8-atp6* and *nad4-nad4l* had a 7 bp ATGATAA pattern. Overall, ten intergenic/non-coding regions are present, ranging in length from 1 to 200 bp (Table 2, Fig. 1).

**Table 2:** Mitochondrial genome organization of *O. laetus.*

Name	Start	Strand	Size (bp)	Anticodon	Start codon	Stop codon	Intergenic nucleotides
*trnI*	1–62	J	62	GAT	–	–	0
*trnQ*	60–128	N	69	TTG	–	–	− 1
*trnM*	128–195	J	68	CAT	–	–	0
*nad2*	196–1185	J	990	–	ATT	TAA	− 2
*trnW*	1184–1246	J	63	TCA	–	–	− 8
*trnC*	1239–1300	N	62	GCA	–	–	0
*trnY*	1301–1362	N	62	GTA	–	–	1
*cox1*	1364–2902	J	1539	–	TTG	TAA	− 5
*trnL2*	2898–2962	J	65	TAA	–	–	0
*cox2*	2963–3637	J	675	–	ATA	AAA	1
*trnK*	3639–3709	J	71	AAG	–	–	0
*trnD*	3710–3775	J	66	GTC	–	–	0
*atp8*	3776–3934	J	159	–	ATT	TAA	− 7
*atp6*	3928–4593	J	666	–	ATG	TAA	− 1
*cox3*	4593–5379	J	787	–	ATG	T	0
*trnG*	5380–5445	J	66	GGA	–	–	0
*nad3*	5446–5799	J	354	–	ATA	TAA	0
*trnA*	5800–5862	J	63	TGC	–	–	0
*trnR*	5863–5925	J	63	TCG	–	–	0
*trnN*	5926–5993	J	68	GTT	–	–	− 1
*trnS1*	5993–6061	J	69	GCT	–	–	− 1
*trnE*	6061–6125	J	65	TTC	–	–	0
*trnF*	6126–6188	N	63	GAA	–	–	− 1
*nad5*	6188–7861	N	1674	–	ATT	TAA	41
*trnH*	7901–7962	N	62	GTG	–	–	2
*nad4*	7965–9284	N	1320	–	ATG	TAA	− 7
*nad4l*	9278–9559	N	282	–	ATT	TAA	2
*trnT_0*	9562–9623	J	62	TGT	–	–	− 1
*trnT_1*	9823–9884	J	62	TGT	–	–	200
*trnP*	9885–9948	N	64	TGG	–	–	1
*nad6*	9950–10,417	J	468	–	ATT	TAA	− 1
*cob*	10,417–11,553	J	1137	–	ATG	TAG	− 2
*trnS2*	11,552–11,620	J	69	TGA	–	–	17
*nad1*	11,637–12,566	N	930	–	ATA	TAA	− 6
*trnL1*	12,561–12,626	N	66	TAG	–	–	− 25
*rrnL*	12,602–13,845	N	1244	–	–	–	30
*trnV*	13,876–13,942	N	67	TAC	–	–	1
*rrnS*	13,944–14,710	N	767	–	–	–	− 1
Control region	14,710–15,672	–	962	–	–	–	–

*J* majority strand, *N* minority strand, − ve overlapping genes, + ve intergenic space.

The gene arrangement was similar to the ancestral arrangement with a significant change in the intergenic space between nad4 and *tRNA-H*, which is reduced to 11 bp. Another striking feature was the duplication of the gene tRNA-threonine, which could have occurred due to anticodon mutation or replication slipping^37^. The displacement of *tRNA-H*, closer to *nad4* and shortening of the 41 bp non-coding region to 11 bp was validated by aligning the concerned region of the assembled data with the Sanger sequencing, and 100% identity was obtained, confirming the displacement as shown in Fig. S2. Lygaeidae have a unique synapomorphy by having an unusual spacer between *tRNA-His* and *nad4* in their mitogenomes^38^. Though this was witnessed in *O. laetus* mitogenome, perhaps on the other end, a feature that is generally expected to be in Lygaeidae.

### Nucleotide composition and codon usage

Nucleotide composition is highly skewed towards A/T, a common feature in mitogenomes^30,31^, and the AT content was 74.22%, and the rest being GC content (25.6%) (Table 3). The standard range for Hemiptera ranges between 69.53–83.53% and 73.15–76.04% in the case of Lygaeoidae (Table 4). The AT skew observed was 0.11, indicating adenines more in number than thymines, and the GC skew of − 0.19 indicates cytosines outnumbering guanines by a whisker.

**Table 3:** Comparison of skewness in other insect species.

NCBI Acc.#	Species	Family	A	G	C	T	Total	AT%	GC%	AT skew	GC skew
*NC_015842.1*	*Agriosphodrus dohrni*	Reduviidae	6422	2007	2569	5472	16,470	72.22	27.78	0.08	− 0.12
*NC_011594.1*	*Acyrthosiphon pisum*	Aphididae	7873	926	1665	6505	16,969	84.73	15.27	0.10	− 0.29
*NC_012446.1*	*Aeschyntelus notatus*	Rhopalidae	6225	1468	2062	4777	14,532	75.71	24.29	0.13	− 0.17
*MT430940.1*	*Aphis gossypii*	Aphididae	7287	924	1676	6158	16,045	83.80	16.20	0.08	− 0.29
*HQ902161.1*	*Apolygus lucorum*	Miridae	6358	1513	1915	4982	14,768	76.79	23.21	0.12	− 0.12
*JX839706.1*	*Chauliops fallax*	Malcidae	7046	1510	2630	4553	15,739	73.70	26.30	0.21	− 0.27
*NC_012449.1*	*Coptosoma bifaria*	Plataspidae	6616	2018	2621	4924	16,179	71.33	28.67	0.15	− 0.13
*JQ739179.1*	*Coridius chinensis*	Dinidoridae	6249	1589	2068	4742	14,648	75.03	24.97	0.14	− 0.13
*NC_020373.1*	*Dolycoris baccarum*	Pentatomidae	6922	1867	2542	5218	16,549	73.36	26.64	0.14	− 0.15
*NC_012421.1*	*Dysdercus cingulatus*	Pyrrhocoridae	7161	1413	2212	5463	16,249	77.69	22.31	0.13	− 0.22
*NC_042436.1*	*Euscopus rufipes*	Pyrrhocoridae	7005	1498	2475	5377	16,355	75.71	24.29	0.13	− 0.25
*NC_022449.1*	*Eusthenes cupreus*	Tessaratomidae	6816	1882	2316	5215	16,229	74.13	25.87	0.13	− 0.10
*NC_012424.1*	*Geocoris pallidipennis*	Geocoridae	6321	1502	2021	4748	14,592	75.86	24.14	0.14	− 0.15
*NC_013272.1*	*Halyomorpha halys*	Pentatomidae	7111	1664	2245	5497	16,517	76.33	23.67	0.13	− 0.15
*NC_012456.1*	*Hydaropsis longirostris*	Coreidae	6871	1514	2541	5595	16,521	75.46	24.54	0.10	− 0.25
*KJ584365.1*	*Kleidocerys resedae*	Lygaeidae	6641	1399	2120	4528	14,688	76.04	23.96	0.19	− 0.20
*MF497725.1*	*Lygaeus sp.*	Lygaeidae	6563	1463	2208	5001	15,235	75.90	24.10	0.14	− 0.20
*EU401991.2*	*Lygus lineolaris*	Miridae	7316	1670	2410	5631	17,027	76.04	23.96	0.13	− 0.18
*NC_012457.1*	*Macroscytus gibbulus*	Cydnidae	6076	1613	2219	4712	14,620	73.79	26.21	0.13	− 0.16
*NC_012458.1*	*Malcus inconspicuous*	Malcidae	6895	1393	2065	5222	15,575	77.80	22.20	0.14	− 0.19
*NC_015342.1*	*Megacopta cribraria*	Plataspidae	6612	1889	2737	4409	15,647	70.44	29.56	0.20	− 0.18
*KX505856.1*	*Neolethaeus assamensis*	Rhyparochromidae	6692	1567	2478	4330	15,067	73.15	26.85	0.21	− 0.23
*JQ806057.1*	*Nesidiocoris tenuis*	Miridae	7253	1943	2442	5904	17,542	75.00	25.00	0.10	− 0.11
*NC_011755.1*	*Nezara viridula*	Pentatomidae	7301	1661	2244	5683	16,889	76.88	23.12	0.12	− 0.15
*MN599979.1*	*Nysius plebeius*	Lygaeidae	7574	1656	2340	5797	17,367	76.99	23.01	0.13	− 0.17
*SRR852651x*	*Oxycarenus laetus*	Lygaeidae	6472	1636	2393	5164	15,665	74.28	25.72	0.11	− 0.19
*KX216853.1*	*Panaorus albomaculatus*	Rhyparochromidae	7258	1555	2364	5168	16,345	76.02	23.98	0.17	− 0.21
*NC_012460.1*	*Phaenacantha marcida*	Colobathristidae	6442	1563	2296	4239	14,540	73.46	26.54	0.21	− 0.19
*NC_012432.1*	*Physopelta gutta*	Largidae	6710	1510	2297	4418	14,935	74.51	25.49	0.21	− 0.21
*NC_046740.1*	*Rhopalosiphum nymphaeae*	Aphididae	7010	908	1543	6132	15,593	84.28	15.72	0.07	− 0.26
*NC_012462.1*	*Riptortus pedestris*	Alydidae	7198	1624	2400	5969	17,191	76.59	23.41	0.09	− 0.19
*NC_012888.1*	*Stictopleurus subviridis*	Rhopalidae	6512	1558	2163	5086	15,319	75.71	24.29	0.12	− 0.16
*NC_002609.1*	*Triatoma dimidiata*	Reduviidae	6913	1901	3275	4920	17,009	69.57	30.43	0.17	− 0.27
*NC_020144.1*	*Urochela quadrinotata*	Urostylididae	6685	1633	2442	5827	16,587	75.43	24.57	0.07	− 0.20
*NC_012823.1*	*Valentia hoffmanni*	Reduviidae	6467	1615	2488	5055	15,625	73.74	26.26	0.12	− 0.21
*NC_012464.1*	*Yemmalysus parallelus*	Berytidae	6629	1620	1974	5524	15,747	77.18	22.82	0.09	− 0.10

**Table 4:** Codon count and relative synonymous codon usage in *O. laetus* mitochondrial PCGs.

Codon	Count	RSCU	Codon	Count	RSCU	Codon	Count	RSCU	Codon	Count	RSCU
UUU(F)	203	− 1.5	UCU(S)	70	− 1.25	UAU(Y)	224	− 1.53	UGU(C)	56	− 1.51
UUC(F)	68	− 0.5	UCC(S)	42	− 0.75	UAC(Y)	68	− 0.47	UGC(C)	18	− 0.49
UUA(L)	310	− 3.22	UCA(S)	93	− 1.66	UAA(*)	248	− 1.56	UGA(W)	86	− 1.51
UUG(L)	44	− 0.46	UCG(S)	11	− 0.2	UAG(*)	70	− 0.44	UGG(W)	28	− 0.49
CUU(L)	81	− 0.84	CCU(P)	87	− 1.43	CAU(H)	71	− 1.3	CGU(R)	13	− 1.13
CUC(L)	23	− 0.24	CCC(P)	63	− 1.03	CAC(H)	38	− 0.7	CGC(R)	8	− 0.7
CUA(L)	99	− 1.03	CCA(P)	80	− 1.31	CAA(Q)	139	− 1.54	CGA(R)	19	− 1.65
CUG(L)	21	− 0.22	CCG(P)	14	− 0.23	CAG(Q)	41	− 0.46	CGG(R)	6	− 0.52
AUU(I)	311	− 1.48	ACU(T)	94	− 1.32	AAU(N)	308	− 1.45	AGU(S)	58	− 1.04
AUC(I)	110	− 0.52	ACC(T)	62	− 0.87	AAC(N)	118	− 0.55	AGC(S)	43	− 0.77
AUA(M)	289	− 1.71	ACA(T)	114	− 1.6	AAA(K)	391	− 1.53	AGA(S)	90	− 1.61
AUG(M)	49	− 0.29	ACG(T)	15	− 0.21	AAG(K)	120	− 0.47	AGG(S)	41	− 0.73
GUU(V)	49	− 1.2	GCU(A)	42	− 1.57	GAU(D)	55	− 1.43	GGU(G)	27	− 1.13
GUC(V)	23	− 0.56	GCC(A)	24	− 0.9	GAC(D)	22	− 0.57	GGC(G)	7	− 0.29
GUA(V)	80	− 1.96	GCA(A)	37	− 1.38	GAA(E)	98	− 1.65	GGA(G)	46	− 1.92
GUG(V)	11	− 0.27	GCG(A)	4	− 0.15	GAG(E)	21	− 0.35	GGG(G)	16	− 0.67

A similar trend was observed with PCGs as well. The PCGs in the J strand have AT skew of − 0.025 and a GC skew of − 0.130. At the same time, PCGs in the N strand have AT skew of − 0.35 and a GC skew of 0.244. They are indicating that J-strand PCGs are much less AT and GC skewed than N-strand genes. The rRNA genes have a GC content of 25.7% and 22.6% for *rrnS* and *rrnL*, respectively. *G. pallidipennis* is found to closely match the statistics obtained for the *O. laetus* when the comparison is made among the Lygaeoidae (Table 3). After analyzing 100 + genomes, Wei et al., reported GC skewness as an indication of *oriC* inversion associated with replication orientation and AT skew dictating gene direction and codon usage, both related to the mitogenome strand asymmetry^39^.

A slight variation in the average codons used among our samples was seen and found 14 bp less in BTI. However, a more extensive analysis revealed that the codon AUU (isoleucine) = 311.0 and GCG (Alanine) = 4.0 were having the highest and lowest frequencies in both the samples (Table 7). Predominant codons like Ile, Met, Phe, and Leu were entirely composed of A and T nucleotides. The nucleotide composition of each codon observed for the entire genome shows that the possibility of A occurring at the first position is 42.4, the second position is 39.0, and the third position is 42.6. It was 33.0, 34.0, and 42.6; 10.4, 10.8, and 9.8; and 15.3, 15.4, and 14.1 for T, G, and C nucleotides, respectively.

**Figure 2:**
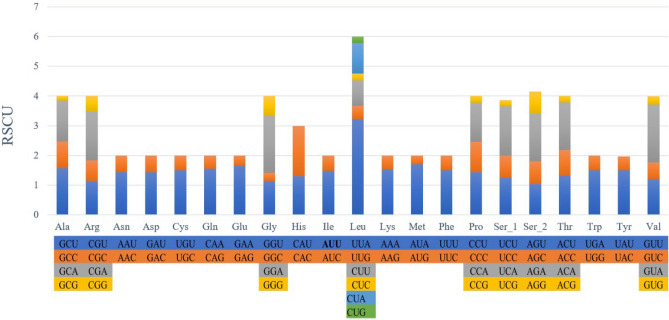
Relative synonymous codon usage.

The relative synonymous codon usage (RSCU) for each amino acid was calculated (Table 4), and a graph was manually generated. It is the number of times a codon is repeated in relation to the uniform synonymous codon usage, i.e., all codons of amino acid have the same chances of appearing in the sequence. It can be observed from Fig. 2 that all the codons of all amino acids have been strictly used, but the distribution is not equal. This distribution can act as markers for identification at the species level and intra-ordinal level.

### Protein coding genes

All the identified 13 PCGs were confirmed for length and sequence by comparing them with the Illumina HiSeq RNA-Seq data generated by our lab for another study (SRR8526511 and SRR8526512). The results obtained from the MITOS2 server agreed with the cross-comparison and verification with the transcriptomic data (results not shown). As shown in Table 2 above, twelve genes have ATN as a start codon, only *cox1* has TTG, which can be seen as a start codon for *cox1* in many other species^40,41^. Four PCGs (*nad2*, *cox2*, *nad3*, *nad1*) have ATA as their start codon, four other genes (*atp8*, *nad5*, *nad4l*, and *nad6*) have ATT as their start codon. The genes *atp6*, *cox3*, *nad4*, *cob* start with codon ATG. Almost all the genes end with the typical non-sense codon, but few genes have incomplete/different termination codons like *cox2* ending with AAA, *cox3* ending with an incomplete codon: T-. The majority of the genes are found on the J-strand with much lower AT and GC Skew. Each PCG was aligned and compared with 35 other species to confirm consensus sequences and significant motifs.

Further, from Table 5, we can infer that there was not a vast difference between AT and CG richness between rRNA, tRNA, and most PCGs. However, the cytochrome family of PCGs *cox1*, *cox2*, *cox3*, and *cob* had a higher representation of GC than the other components of the mitogenome. These genes had an average of 30% GC content which was much lower, 17–26% for other PCGs. The pattern followed by the cytochrome family of genes was also evident for the control region. It has been known that the cytochrome family of genes, primarily, *cox1* and *cob*, are used as markers for eukaryotic species identification and evolutionary studies. The higher incidence of GC in these PCGs may well be attributed to being less prone to environmental mutations, codon usage, and therefore, species identity is maintained^42^.

**Table 5:** Nucleotide composition and skewness of the mitogenome of *O. laetus.*

*O. laetus*	Total size (bp)	A	T	G	C	GC%	AT%	AT Skew	GC Skew
mtgenome	15,672	6471	5161	1633	2389	25.664	74.222	0.117	− 0.188
*nad2*	990	381	407	91	111	20.404	79.596	− 0.033	− 0.099
*cox1*	1539	492	553	222	271	32.034	67.901	− 0.058	− 0.099
*cox2*	675	249	223	86	117	30.074	69.926	0.055	− 0.153
*atp8*	159	71	54	11	23	21.384	78.616	0.136	− 0.353
*atp6*	666	244	245	71	106	26.577	73.423	− 0.002	− 0.198
*cox3*	787	285	270	106	126	29.479	70.521	0.027	− 0.086
*nad3*	354	136	129	36	53	25.141	74.859	0.026	− 0.191
*nad5*	1674	430	857	234	152	23.059	76.882	− 0.332	0.212
*nad4*	1320	315	680	202	122	24.545	75.379	− 0.367	0.247
*nad4l*	282	64	153	47	18	23.050	76.950	− 0.410	0.446
*nad6*	468	173	214	34	47	17.308	82.692	− 0.106	− 0.160
*cob*	1137	376	438	141	182	28.408	71.592	− 0.076	− 0.127
*nad1*	930	232	468	142	88	24.731	75.269	− 0.337	0.235
tRNA genes	1497	575	547	215	159	24.983	74.950	0.024	0.150
rRNA genes	2011	659	873	301	178	23.819	76.181	− 0.140	0.257
Control region	962	328	336	93	202	30.665	69.023	− 0.012	− 0.369

### Non-coding regions

The control region is the largest functional non-coding entity in the mitogenomes, with a length of 962 bp. There are four major spacers spread throughout the genome, ranging from 17 to 200 bp. Small gaps of 1–3 bp were also observed between the genes. The important rearrangement region of 41 bp is found between *nad5* and *trnH*. The second-largest gap measuring 200 bp in length is found between *trnT* replicates (*trnT_0* and *trnT_1*), other small intergenic spaces were found between *trnS2* and *nad1*, *rrnL* and *trnV* with lengths of 17 and 25 bp, respectively.

The spacer between *trnS* and *nad1* is common in most insects. The conserved sequence block (CSB) region has two consensus regions, indicating that tandem duplication and random loss (TDRL) process could be a common intermediate step^37,43^. Two consensus motifs are 5 bp long AATGA and ACCTA sequences.

The control region is the major non-coding spacer, considered the box of origin sites for replication and transcription^44^. The largest crucial non-coding region is also helpful in species identification. Due to its high divergence, the evolution of tandem repeats, and variability, it can be used as markers, making it a potential species-specific marker^11^. Studies about the control region in Echinoides have shown that due to the region's unique features, it has high compatibility across the entire class, outperforming conventional mitochondrial markers^45^. Of all the arthropods sequenced, a few prevalent motifs were noted: (1) Tandem/microsatellite repeats (Tables 6, 7), (2) Poly-thymine sequence, (3) a AT-rich region, (4) A stem-loop structure^15^. We identified all these motifs in our mitogenomes. Some additional striking features were also seen in other insects. Two types of tandem repeats were identified with a consensus size of 22 copy number 2 and another with size 20 and copy number 3 spread across the genome except in the control region.

**Table 6:** Microsatellites in the control region.

Position	Length	Repeats	Sequence
14,754	2	3	CCCCCC
14,848	2	3	TATATA
14,858	2	5	TATATATATA
14,963	4	3	ATTTATTTATTT
15,196	2	3	CCCCCC
15,259	2	3	CACACA
15,353	2	3	AAAAAA

**Table 7:** Tandem repeats in the mitogenome of *O. laetus.*

Indices	Period size	Copy number	Consensus size	Percent matches	Percent indels	Score	A	C	G	T	Entropy (0–2)	Consensus seq
5756–5799	22	2	22	91	8	72	54	2	9	34	1.44	TATATTAGAATGAACTAAATAA
13,503–13,564	22	3	20	79	11	70	54	1	0	43	1.09	TAATTATAAATTAAATTTAA

### tRNAs and rRNAs

As mentioned earlier, we identified 23 tRNAs in the *O. laetus* mitogenome rather than the regular 22, lengths ranging from 60 to 72 bp. The structures predicted were clover-leaf or isoforms with a few exceptions: *trnS1*(AGN), in which the DHU arm (dihydrouridine) was reduced to just a loop of 6 bp, which is the case for most metazoans (Fig. S3)^46^. The *trnV* had formed two loops in both the DHU and TѱC arms. A duplicate of *trnT_0* was identified as *trnT_1*, which has a similar structure to *trnS1* and resembled its isoform due to the reduction of the TѱC arm to a loop, though the sequence was highly similar to *trnT_0*. These features proved that this novel gene order could be explained by the tandem duplication/random loss (TDRL) model^37^. The wobble and mismatch pairs common in insects' tRNA were corrected by comparing data from two software MITOS2^24^ and tRNAScan-SE^47^.

Keeping with the trend of mitogenome patterns, two rRNA genes were identified and further aligned with 35 other species to confirm the lengths and motifs. The large subunit gene of rRNA was 1244 bp in length and positioned between *trnV* and *trnL-1*. The GC content was about 22.6%, and the gene is AT-rich. The smaller subunit gene of rRNA was 767 bp in length with a slightly higher GC content of 25.8%.

### Phylogenetic analysis

We used only one *O. laetus* (CBE) mitogenome to maintain equality with the 35 other species' mitogenomes and avoid any skewness/influence of our mitogenome on the final tree. Seven different super-families chosen in this study belonged to Hemiptera (Aphidoidea, Cimicoidea, Reduvioidea, Pentatomoidea, Coreoidea, Pyrrhocoroidea, and Lygaeoidea). Hemiptera is one of the largest and diverse groups of insects which has more than 110 genera. GAMMA + P-Invar model parameter estimates were based on the 13,883 alignment patterns with the help of the GTR substitution matrix, and the overall tree length is 16.016.

**Figure 3:**
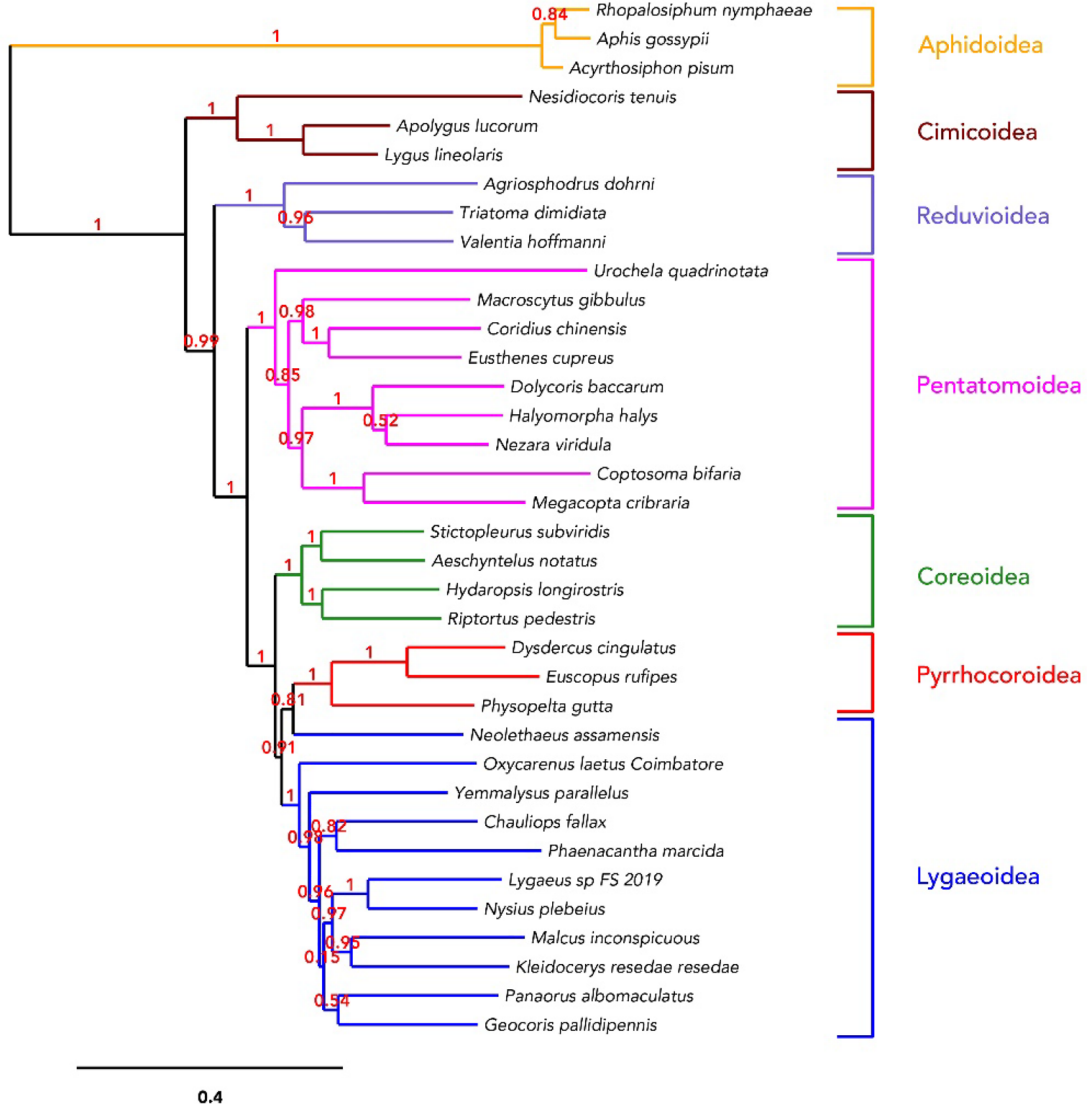
Phylogenetic relationship inferred among 36 whole mitochondrial genomes from seven different super-families of Hemiptera. The above tree shows *O. laetus* and other member species are clustered under their respective super-families.

In the phylogenetic tree shown in Fig. 3 and Fig. S4 (Bayesian inference method), it can be seen that all the species are well placed under their respective super-families. All the 11 Lygaeoidea super-family species have found themselves accommodated in a polyphyletic clade, including species from the Lygaeidae to which *O. laetus* belongs. Studies have confirmed that Lygaeidae is polyphyletic^48^, meaning evolution from several common ancestors. Incidentally, scientists have differed over the years on the lineage of Lygaeidae. They have concluded the need for further studies to reach a consensus on this, as the super-family Lygaeoidea consists of several sub-families within itself.

*O. laetus* remains a separate member among the selected Lygaeoidea species closely related to *Y. parallelus* (Berytidae), resulting in *Lygaeus Sp.,* and *N. plebeius* in one clade and *M. inconspicuous* and *K. resedae resedae* in the other. The rigorous phylogenetic analysis supported by higher branch confirmation values only confirms the clustering and relationships already documented and established. In our earlier study^49^, we had identified the species and studied its phylogenetic relationship with other species using COI sequences. Also, the clustering of *O. laetus* in the Lygaeoidea only confirms this study's sequencing, assembly, and annotation accuracy. However, there always will be opportunities for betterment. Since there are no similar prior mitogenomic studies in Lygaeoidea/Lygaeidae, we could not compare our results. Nevertheless, results obtained from this study will make a significant contribution to future studies involving the Lygaeidae family under the Lygaeoidea super-family.

## Discussion

The species *O. laetus,* although a secondary pest of the cotton plant, is a severe threat to the overall quality and quantity of cotton produce. The insect is widely distributed in various parts of Asia irrespective of climate and soil conditions. The emphasis of this study was to sequence and annotate the complete mitogenome to understand if the two species from these locations are different from each other that might be rendering higher pesticide resistance in BTI (N. India). As both the BTI and CBE samples essentially have identical mitogenomes, the species are the same. Hence, the probability of mutation in the detox system could be assumed as the reason for its survival under such relatively high pesticide and harsh conditions, which the gene expression studies can establish.

The mtDNA is responsible for various essential physiological functions, primarily oxidative phosphorylation^50^. Genes such as NAD and Cytochrome oxidase play vital roles in the ETC pathway^51^. Although the gut microbes are primarily responsible for digestion of harmful pesticides, it works in association with the gut cells for the ultimate goal of respiration^52^. Therefore, the mitochondrial genes are indirectly involved in the digestion of pesticides and insecticides. Targeting these genes and other pathways, such as P450, GST, ABC, and others, can help eradicate pests without reliance on harmful chemicals^53,54^. There has been massive research on mtDNA of insects due to its smaller size, comparative and phylogenetic analysis being the majority^55^. The order Hemiptera also has such comparative and phylogenetic studies^56^. This provides data on intra-ordinal and inter-ordinal relations amongst different insect species. The Lygaeidae family under this order comprises many important pests and species, yet only four have completely sequenced and analysed mitogenomes^57^. This type of analysis provides a perspective for other research and even helps understand evolution to some extent.

In this study, the mitochondrial genome of *O. laetus* has been sequenced, thoroughly analysed, annotated, and compared with 35 other species from Hemiptera. The gene order is similar to the ancestral arrangement except for a few rearrangements. The genome organization was similar to almost all Hemipterans. Unlike the typical 37 genes, 38 genes were determined; this included 13 PCGs, 23 tRNAs, and 2 rRNAs. The gene order was compared with other species from other genera also. There were 16 overlaps observed between the genes which were more or less similar to other species. The highest was 25 bp obtained between *trnL1* and *rrnL*. Another conserved overlap of 7 bp was observed between *atp8* and *atp6*, and *nad4* and *nad4l* were also observed in Melon thrips^58^. The control region is the largest non-coding region obtained.

Almost all the PCGs started with the start codon ATN; only the *cox1* gene has TTG as its start codon, again observed in other species as well^41^. The genes end with complete stop codons except for *cox2* and *cox3*. All the features obtained concerning the PCGs are ancestral and do not vary much from what is already known. The codon usage, AT, and GC skews were all similar to the range obtained for Hemipterans.

Some striking differences have been found in both samples, but deflect from the common are: one of them is the duplication of tRNA-Threonine, which increased the number of tRNAs to 23. The duplicates are labeled as *trnT_0* and *trnT_1*. The duplicate has a similar structure to *trnS1* and resembled its isoform due to the reduction of the TѱC arm to a loop^37^. Similar observations were seen in *Reduvius tenebrosus*^59^*.* This novel rearrangement has been explained by the tandem duplication/random loss (TDRL) model. All the other tRNA structures predicted were clover-leaf, with the above two exceptions^60^. This feature has not been observed for previously sequenced mitogenomes but is seen for newly annotated ones, hinting at mutation and evolution.

The second striking difference observed was the gene rearrangement of the intergenic space between *trnH* and *nad4*, which is considered a synapomorphy of Lygaeoidea, which was reduced to 11 bp, as opposed to 41 bp in the same super-family.

The control region is relatively smaller in length with 962 bp. All the standard features of the control region, similar to the study of *C. fallax,* were observed except for the tandem repeats^61^. Microsatellite repeats replacing the tandem repeats were also observed in various species such as *Panaorus albomaculatus*^36^*.*

## Conclusion

The taxonomy of Hemiptera has always put up several challenging questions, and researchers across the globe have done extensive studies. The emphasis of this study was to find if there exist another cryptic sister species in the two extreme regions of India—Coimbatore, and Bhatinda, where cotton crop scientists witnessed different levels of bug infestation. As the study concludes, the mitogenomes were 99.9% identical and gave us an understanding of their presence in these two places. The mitogenome sequenced provides many genetic markers from PCGs to control regions for identification purposes.

Our study is the first detailed mitochondrial genomic study in the genus *Oxycarenus*. We also have found some novel rearrangements in the mitogenome, which will help understand the genus's evolution and ecology. The results obtained from this study, along with the whole genome of this species when available, would provide possibly new and exciting prospects that could help in biocontrol and mitigating resistance of this economically important species. From the resistance to pesticide perspective, further studies involving whole transcriptomic approaches may lead to vital discoveries. Therefore, the data and the findings from this study would further help in the evolutionary studies of Oxycarenidae, Lygaeidae, and Hemiptera in the future.

## Supplementary Information

Supplementary Information.

## Abbreviations

mtDNA: Mitochondrial DNA
BTI: Bhatinda
CBE: Coimbatore
