# Supplementary material for: Complete mitochondrial genome sequencing of Oxycarenus laetus (Hemiptera: Lygaeidae) from two geographically distinct regions of India
Source: Sci Rep. 2021 Dec 9;11:23738. doi: 10.1038/s41598-021-02881-0 (PMC8660866; doi:10.1038/s41598-021-02881-0)
Supplement: Supplementary file 1 — Supplementary Information. [file 41598_2021_2881_MOESM1_ESM.docx]

**Complete mitochondrial genome sequencing of *Oxycarenus laetus* (Hemiptera: Lygaeidae) from two geographically distinct regions of India**

Shruthi Chalil Sureshan^1^., Ruchi Vivekanand Tanavade^1^., Sewali Ghosh^2^., Saswati Ghosh^3^., Raja Natesan Sella^4^., Habeeb Shaik Mohideen^1^**^*^**

^1^Bioinformatics and Entomoinformatics Lab, Department of Genetic Engineering, School of Bioengineering, SRM Institute of Science and Technology, Kattankulathur, Tamilnadu – 603203, India.

^2^Department of Advanced Zoology and Biotechnology, Guru Nanak College, Chennai, Tamilnadu – 600042

^3^Department of Virology, King Institute of Preventive Medicine and Research, Chennai, Tamilnadu – 600032

^4^Membrane Protein Lab, Department of Genetic Engineering, School of Bioengineering, SRM Institute of Science and Technology, Kattankulathur, Tamilnadu – 603203, India

**^*^**Corresponding author, Email: [habeebm@srmist.edu.in](mailto:habeebm@srmist.edu.in) &[habeeb_skm@yahoo.co.in](mailto:habeeb_skm@yahoo.co.in)

**Supplementary data**


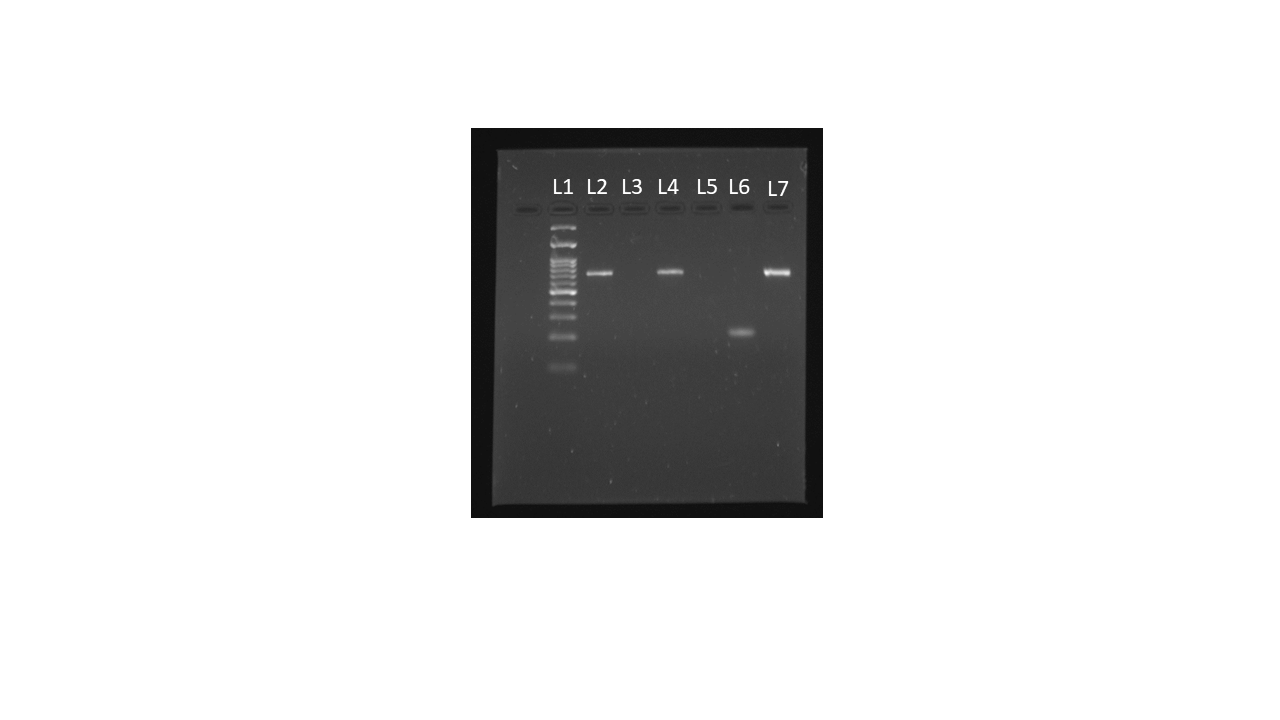


**Figure S1: Validation of mitochondrial DNA by amplification of COI (mitochondrial gene) and H4 (nuclear gene).** L1: 100 bp ladder; L2 & L4: PCR product of COI gene in sample BTI and CBE; L3 & L5: no amplification of H4 gene in mitochondrial DNA in sample BTI and CBE respectively; L6: COI gene amplified from genomic DNA (control); L7: H4 gene amplified from genomic DNA (control)


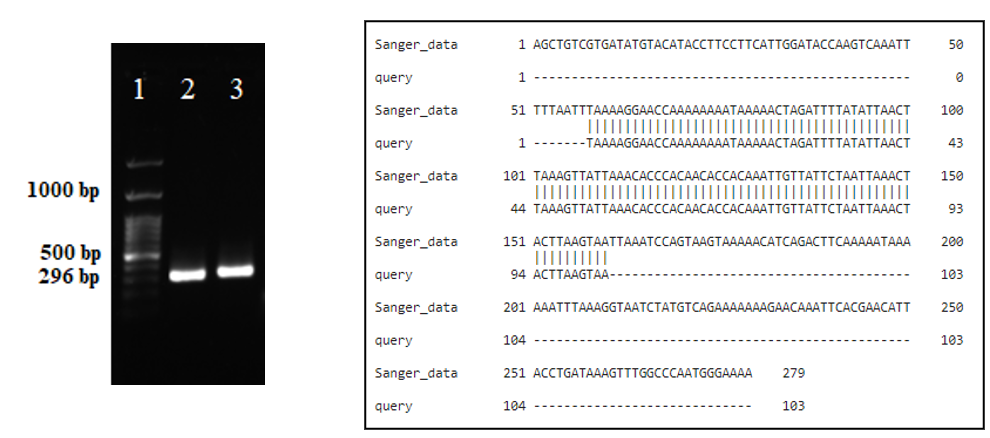


**(a) (b)**

**Figure S2: (a) PCR product of NAD5 and trnH gap was run on 1% gel. (b)Alignment showing 100% identity between the sequenced and rearranged 41 bp.**

(a): Lane 1: 100 bp DNA ladder, Lane 2: PCR product of BTI sample, and Lane 3: PCR product of CBE sample.

(b) The first 41 bases of the match represent the gap, and the following 72 codes for tRNA_H confirms that the gap lies before the tRNA_H gene and has been displaced.


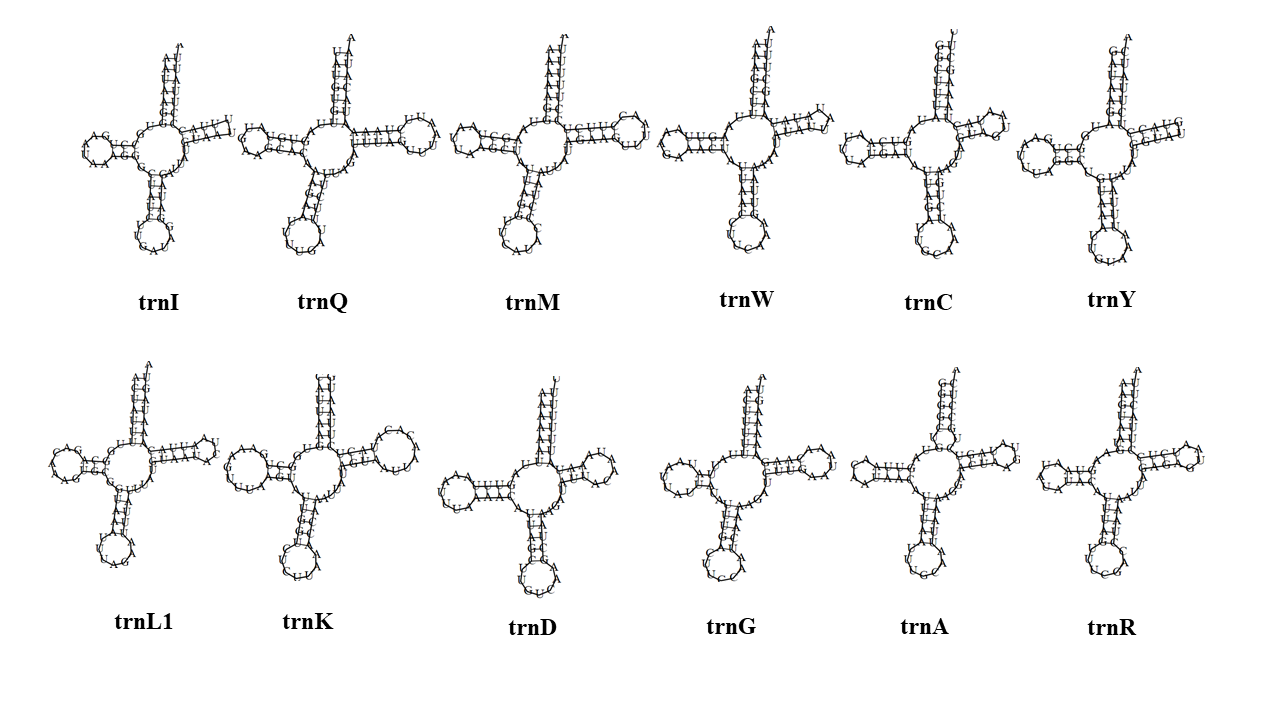


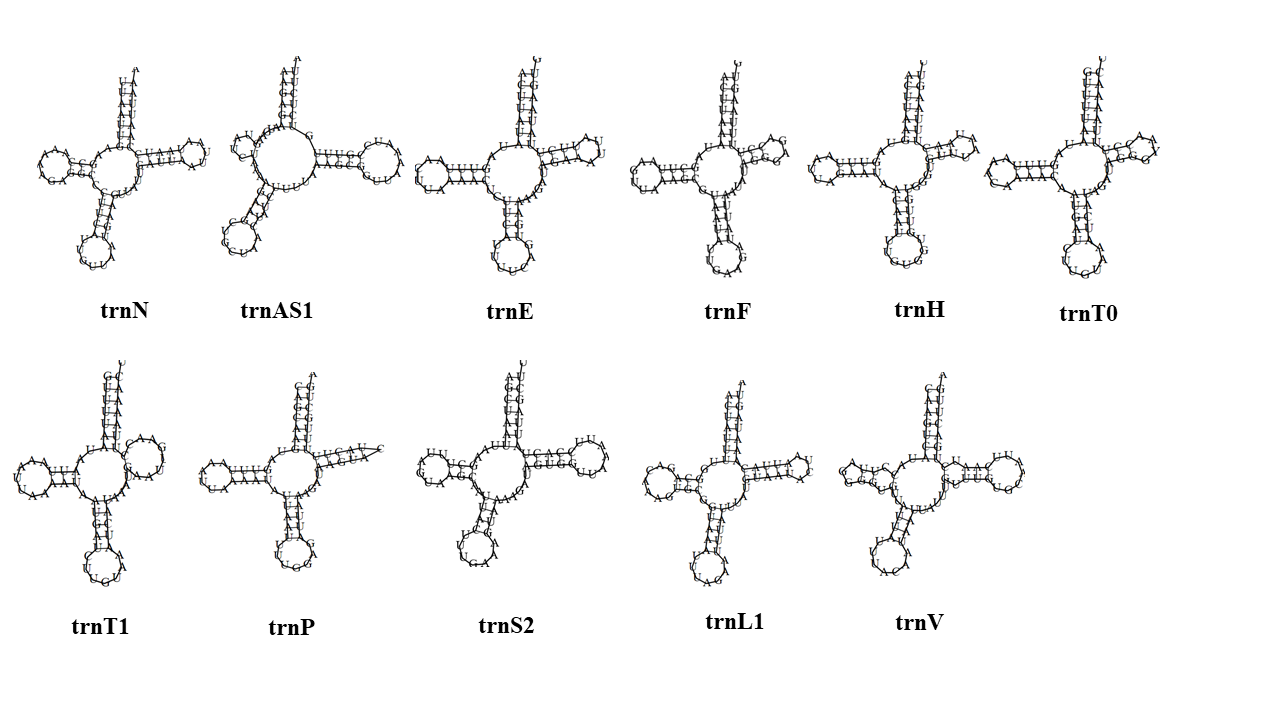


**Figure S3: Secondary structures of tRNAs**

Secondary structures of tRNAs predicted with the help of tRNAScan-SE. All the tRNA have folded into near clover-leaf models, except trnS1 and trnV.


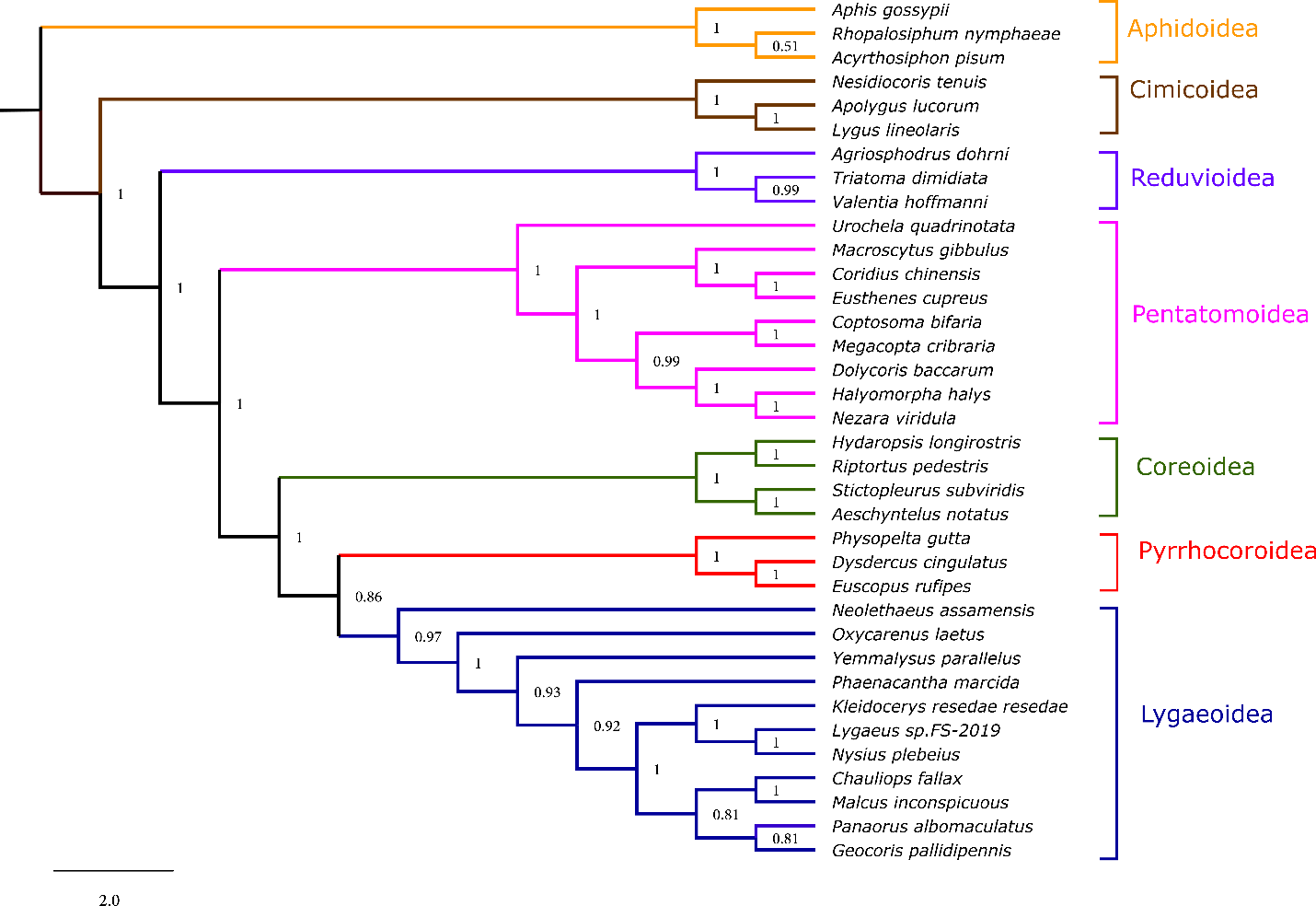


**Figure S4**: Phylogenetic tree of 36 mitochondrial genome constructed using MrBayes.
